# *In vitro* trackable assembly of RNA-specific nucleocapsids of the respiratory syncytial virus

**DOI:** 10.1074/jbc.RA119.011602

**Published:** 2019-12-10

**Authors:** Yunrong Gao, Dongdong Cao, Hyunjun Max Ahn, Anshuman Swain, Shaylan Hill, Claire Ogilvie, Matthew Kurien, Taha Rahmatullah, Bo Liang

**Affiliations:** Department of Biochemistry, Emory University School of Medicine, Atlanta, Georgia 30322

**Keywords:** negative-strand RNA virus, RNA–protein interaction, chromatography, EM, protein assembly, genome replication, nucleocapsid (NC), nucleocapsid-like particle (NCLP), nucleoprotein (N), respiratory syncytial virus (RSV)

## Abstract

The templates for transcription and replication by respiratory syncytial virus (RSV) polymerase are helical nucleocapsids (NCs), formed by viral RNAs that are encapsidated by the nucleoprotein (N). Proper NC assembly is vital for RSV polymerase to engage the RNA template for RNA synthesis. Previous studies of NCs or nucleocapsid-like particles (NCLPs) from RSV and other nonsegmented negative-sense RNA viruses have provided insights into the overall NC architecture. However, in these studies, the RNAs were either random cellular RNAs or average viral genomic RNAs. An in-depth mechanistic understanding of NCs has been hampered by lack of an *in vitro* assay that can track NC or NCLP assembly. Here we established a protocol to obtain RNA-free N protein (N^0^) and successfully demonstrated the utility of a new assay for tracking assembly of N with RNA oligonucleotides into NCLPs. We discovered that the efficiency of the NCLP (N–RNA) assembly depends on the length and sequence of the RNA incorporated into NCLPs. This work provides a framework to generate purified N^0^ and incorporate it with RNA into NCLPs in a controllable manner. We anticipate that our assay for *in vitro* trackable assembly of RSV-specific nucleocapsids may enable in-depth mechanistic analyses of this process.

## Introduction

Nonsegmented negative-sense (NNS)^2^ RNA viruses include many significant human pathogens, such as rabies, Ebola, and respiratory syncytial virus (RSV) (1). Unfortunately, no effective vaccine or antiviral therapy is available to prevent or treat these pathogens, including RSV (2, 3). Since its first isolation in 1955, RSV infection has been the leading cause of severe lower respiratory tract diseases in young children, old adults, and immunocompromised people worldwide (4, 5).

**Figure 1.**
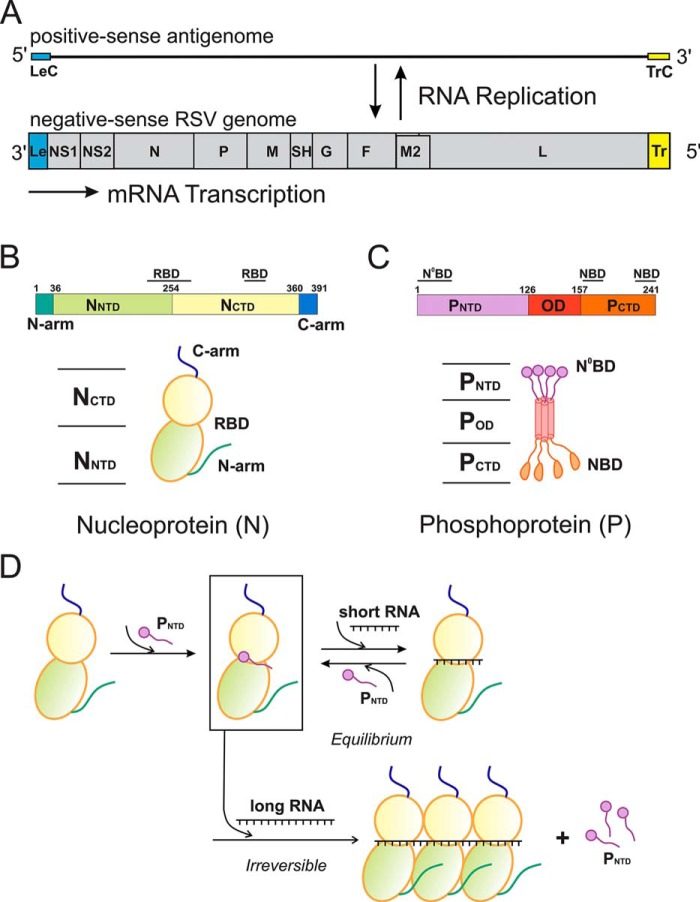
***In vitro* reconstitution of NCLPs (N–RNA).** *A*, the negative-sense RSV genome and its positive-sense antigenome. The RSV genome is depicted from 3′ to 5′, showing the Le, 10 viral genes (NS1, NS2, N, P, M, SH, G, F, M2, and L), and the Tr regions. The Le and TrC regions contain essential *cis*-acting signals for RNA synthesis. *B*, schematic and cartoon view of the nucleoprotein. *RBD*, RNA-binding domain. *C*, schematic and cartoon view of the phosphoprotein. *D*, P_NTD_ is used as the chaperone of N to generate soluble monomeric N^0^P (*box*). Then P_NTD_ is removed and assembled with the desired length and sequence of RNA to form N–RNA.

The RSV genome contains 10 genes that encode 11 proteins. The RSV genome (negative-sense) RNA acts as the template for transcription to synthesize 10 viral mRNAs as well as the template for replication to produce antigenome (positive-sense) RNA. The antigenome RNA, in turn, serves as the template to generate genome RNA (Fig. 1*A*). Both the genome and antigenome RNAs are encapsidated entirely by the nucleoprotein (N) to form helical nucleocapsids (NCs) during RNA synthesis, and each RSV N protein covers 7 nucleotides (nt) (1, 6–9). For example, the length of the RSV genome (A2 strain) is 15,222 nt, which requires more than 2,100 copies of N to coat the entire genome or antigenome. The NCs remain mostly intact during RNA synthesis. It is thought that several N proteins transiently dissociate from the RNA template to allow access of the polymerase and rebind to RNA after synthesis (10, 11).

RSV shares a common strategy for genome replication and gene expression with all NNS RNA viruses (8, 12). During transcription, the polymerase uses a single promoter in the 3′ terminus of the genome and initiates and terminates mRNA transcription responding to gene start (GS) and gene end (GE) signals, respectively. During replication, the polymerase initiates at 3′ ends of genome or antigenome RNAs and ignores all GS and GE signals to synthesize a full-length complementary RNA. The 44-nt leader (Le) at the 3′ end of the genome and 155-nt trailer complementary (TrC) at the 3′ end of the antigenome serve as promoters for RNA synthesis (13, 14) (Fig. 1*A*).

Although the RSV polymerase itself and the RNA synthesis assay have been reconstituted *in vitro* (15), its biologically relevant N–RNA template poses a significant technical hurdle, mainly because the N protein binds nonspecifically to cellular RNAs to form nucleocapsid-like particles (NCLPs) when N is recombinantly expressed (9, 16, 17). Previous structural studies of NCLPs from RSV and other NNS RNA viruses, such as vesicular stomatitis virus and Ebola virus, provided insights into the overall architecture of NCs (9, 17–20). However, in these studies, the RNAs were either random cellular RNAs or average viral genomic RNAs. Therefore, there is a critical need to establish an *in vitro* assay that can track the assembly of virus-specific NCLPs for in-depth mechanistic analysis.

Obtaining a sufficient quantity of recombinant RNA-free N (N^0^) is the first step to reconstitute a trackable NC *in vitro*. The crystal structure of the RSV N–RNA (nonviral) pseudoring revealed that the N protein has two core lobes, with the RNA bound in the central groove (9, 21). Both the N-terminal motif (N-arm) and the C-terminal motif (C-arm) connect the adjacent subunits in the RNA-bound ring, providing a significant stabilizing interaction (9). The N-arm of the N protein is inserted into the compact fold of its adjacent N protein when forming an oligomer of N–RNA. The C-arm of the N protein inhibits premature RNA uptake and prevents incorporation of cellular RNA during expression of N protein (Fig. 1*B*).

The RSV phosphoprotein (P) exists as a homotetramer with a tetramerization domain and is intrinsically flexible in both N- and C-terminal domains (Fig. 1*C*). Previous studies suggested that P not only binds to N^0^ monomers and delivers them to nascent RSV RNA genomes or antigenomes but also interacts with N–RNA to bridge the polymerase to the RNA template (22). Specifically, the N-terminal domain of P (P_NTD_) interacts with N^0^, and the C-terminal domain of P (P_CTD_) interacts with assembled N–RNA (23, 24). Structures of the N^0^–P complex of human metapneumovirus, measles (MeV), Nipah virus, and vesicular stomatitis virus suggest a potential chaperone role of P_NTD_, preventing N from self-aggregating or binding to cellular RNA (11, 21, 22, 25–27).

In this study, we demonstrated the feasibility of large-scale preparation of N^0^ and *in vitro* assembly of trackable NCLPs. We used P_NTD_ as a chaperone to prevent nonvirus-specific N–RNA interactions by coexpressing P_NTD_ and N together, and we established a protocol to obtain large-scale soluble RSV N^0^–P complex. We showed that the purified N^0^–P_NTD_ could be stimulated and assembled into NCLPs by adding RSV-specific RNA oligonucleotides and that P_NTD_ is removed upon addition of RNA (Fig. 1*D*). We then used size exclusion chromatography (SEC) and negative stain EM to characterize and visualize the resulting NCLPs. We examined the length preference and nucleotide selectivity of the RNA in NCLP assembly. We discovered that NCLP assembly with purified N^0^ depends on the length and sequence of the RNA. We also found that, although both length and sequence are critical for assembly of NCLPs, the longer length of RNA can compensate for the efforts of nonpreferred nucleotide-type RNA in NCLP assembly. We identified that the shortest poly(A) RNA that could stimulate assembly of N–RNA is 7 nt. To understand initial NCLP assembly, we examined the position specificity of the first 7 nt using various 7-nt RNA oligos. This study established a powerful tool to further perform in-depth mechanistic studies of RSV RNA synthesis; in particular, *cis*-acting RNA signals and *trans*-acting viral proteins.

## Results

### Purification of N^0^ protein

**Figure 2.**
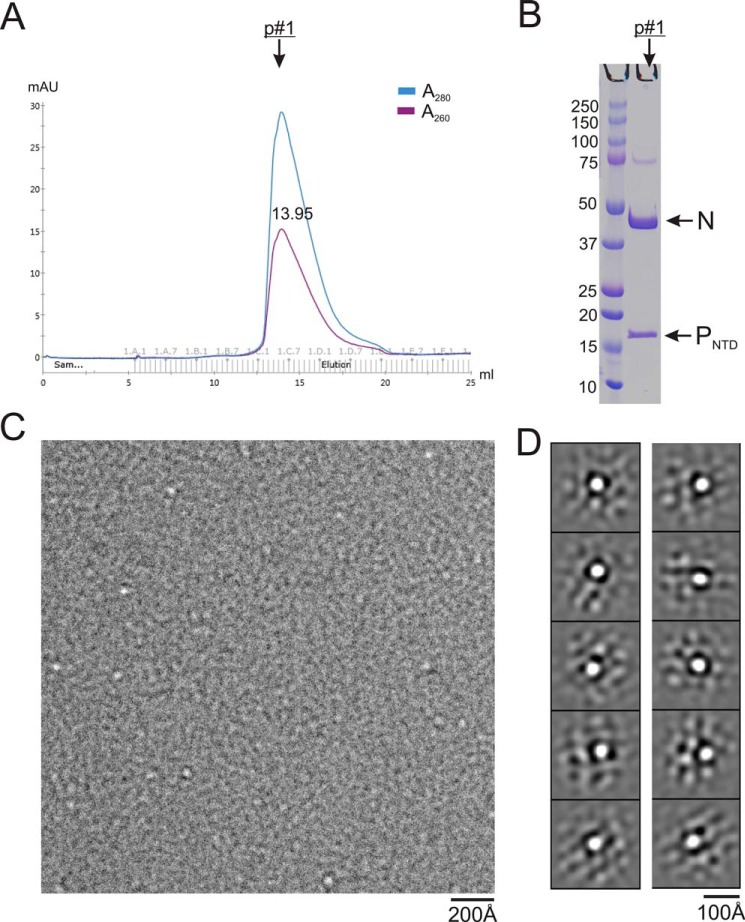
**Preparation of the N^0^–P_NTD_ complex.** *A*, the size exclusion chromatography profile shows one major peak for N^0^–P_NTD_. *A*_260_/*A*_280_ shows that the sample is protein. *B*, *right*, Coomassie-stained SDS-PAGE of the corresponding peak fraction (*p#1*). The nucleoprotein and P_NTD_ are indicated by *arrows. C*, representative negative stain EM image of purified N^0^–P_NTD_. The *white dots* are N^0^–P_NTD_ particles. *D*, class averages of the negative stain EM images, showing the N^0^–P_NTD_ complex with the proper size.

In this study, we established a protocol to obtain large-scale soluble heterodimeric RSV N^0^–P complex. We coexpressed the N and P_NTD_ proteins with a His_10_ tag on the N protein in *Escherichia coli*. The binding of P and RNA to N protein seemed competitive, and we usually obtained a mixed population of N–RNA and N^0^–P_NTD_. We optimized the purification strategy to shift the equilibrium and copurified higher yields of N^0^–P_NTD_. We further purified N^0^–P_NTD_ using ion exchange and SEC (Fig. 2*A*). We showed, using SDS-PAGE, that N^0^–P_NTD_ (peak 1) contained both N and P_NTD_ but did not contain RNA, based on the UV absorbance *A*_260_/*A*_280_ ratio (Fig. 2, *A* and *B*). We also imaged the purified N^0^–P_NTD_ (peak 1) using negative stain EM, and the particles appeared as single white dots on a gray background (Fig. 2*C*). The class averages showed soluble N^0^–P_NTD_ complex as white dots ∼50 Å in diameter (Fig. 2*D*).

### Assembling NCLPs by adding RSV-specific RNA

We then demonstrated that the purified N^0^–P_NTD_ could be stimulated and assembled into NCLPs by adding RSV-specific RNA oligos. We assembled NCLPs by incubating N^0^–P_NTD_ with an excess molar ratio of RNA (the molar ratio of protein:RNA is 1:1.5) and isolated the NCLPs (N–RNA) by running gel filtration chromatography. When the N–RNA complex formed, we observed the appearance of peak 0 (N–RNA) and reduced levels of peak 1 (N) and peak 2 (RNA) in the SEC profile. Depending on whether N–RNA formed and the efficiency of assembly, we could evaluate and compare the value of peak 0 alone and the ratio of (peak 0)/(peak 2). The same procedure applied to other N–RNA assemblies in this study.

**Figure 3.**
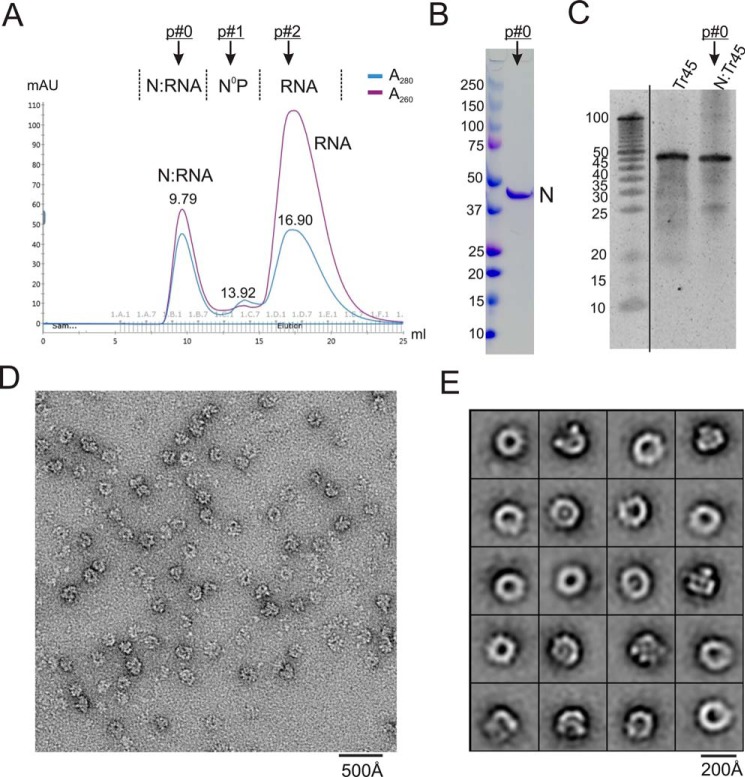
***In vitro* assembly of N–RNA using RNA oligos.** *A*, gel filtration SEC profile of the assembly of N–RNA. Shown are peaks 0, 1, and 2, with *arrows* indicating N–RNA, N, and RNA, respectively. The *A*_260_/*A*_280_ of each peak reflects the sample property of protein–RNA, protein alone, and RNA alone. *B*, SDS-PAGE gel showing that peak 0 is the N–RNA complex (no P). *C*, RNA denaturing gel showing that RNA from peak 0 contains intact length of RNA (45 nt) compared with the RNA-alone control. The excised lanes are indicated by a *vertical line. D*, representative negative stain EM image of an assembled N–RNA complex. The ring-shaped particles with spikes are NCLPs (N–RNA). *E*, class averages of the negative stain EM images, showing the majority of classes of ring-shaped particles.

For example, in the case of N–Tr45, we observed the appearance of peak 0 (N–Tr45) and an almost depleted level of peak 1 (N) (Fig. 3*A*). N–RNA formation (peak 0) was also confirmed by the disappearance of the P protein (P_NTD_) band in the SDS-PAGE gel (Fig. 3*B*), and phenol extraction followed by RNA PAGE denaturing gel showed that Tr45 RNA in complex with N was intact compared with RNA alone (Fig. 3*C*). As shown in the raw micrograph, the larger complexes (N–RNA) formed upon RNA addition (Fig. 3*D*). Class averages revealed that most classes were ring-shaped particles resembling the N–RNA pseudoring structure (Fig. 3*E*) (9, 28). We also used Le44 and LeC44, both of which formed peak 0 (N–RNA), and the negative stain EM images showed similar ring-shaped particles (Fig. S1).

### Length preference of NCLP assembly

**Figure 4.**
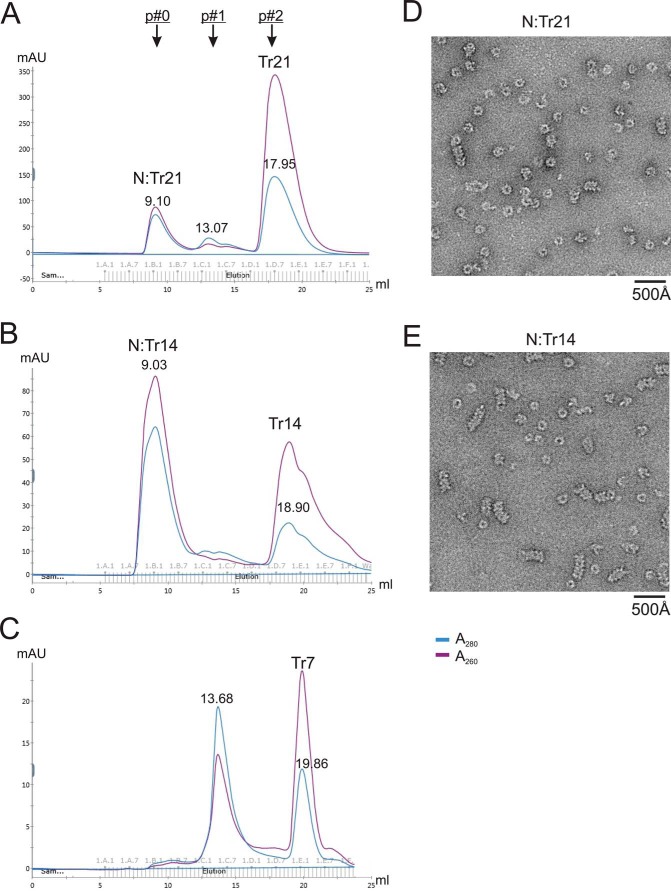
**Length dependence of N–RNA assembly.** *A–C*, gel filtration SEC profiles of the assembly of N–RNA using 21-nt, 14-nt, and 7-nt trailer sequences (Tr21, Tr14, and Tr7), respectively. Tr21 and Tr14 formed peak 0 (N–RNA) but not Tr7. *D* and *E*, representative negative stain EM images of assembled N–RNA complex. Most of the particles are similar to that in Fig. 3, and a portion of particles formed short and curved filaments.

We then examined the length preference of NCLP assembly. We used RNAs of different lengths and sequences to incubate with purified N^0^–P_NTD_. The results were analyzed using gel filtration SEC and negative stain EM. We used 21 nt, 14 nt, and 7 nt of the RSV genomic trailer sequence (Tr21, Tr14, and Tr7) and found that only Tr21 and Tr14 formed stable complexes with N (peak 0) but not Tr7 (Fig. 4, *A–C*). The negative stain EM images of peak 0 of N–Tr21 and N–Tr14 revealed that most of the particles were similar to the particles shown in Fig. 3 (Fig. 4, *D* and *E*).

It is interesting that the *A*_260_/*A*_280_ ratio was slightly higher for N–Tr14 (86.27 mAU/64.20 mAU = 1.34) than for N–Tr21 (87.51 mAU/73.08 mAU = 1.20). The crystal structure of the N–RNA complex suggested that every N protein binds to 7 nt of RNA (9). Theoretically, if binding of N starts at position 1 of RNA, and it is continuously packed, then the *A*_260_/*A*_280_ ratio should have a similar value for 2N:14mer and 3N:21mer. However, exactly how N interacts with RNA and how many copies of N bind with RNA at the initiation steps remain unclear. It is possible that the first N-binding site on RNA is not at position 1 but, rather, at a later position (such as 2, 3, or 4). In that case, because of the space restraint, for the 14-nt RNA, only one N protein is allowed to bind to RNA, but for 21-nt RNA, there will be two N proteins that are allowed to bind to RNA. This may explain a higher *A*_260_/*A*_280_ ratio of N–Tr14 (1N:14mer) than of N–Tr21 (2N:21mer).

To validate the observation of length preference, we also used 21-nt, 14-nt, and 7-nt RNA sequences from the SH gene (derived from the RSV RNA genome): SH21, SH14, and SH7. It showed similar results; SH21 and SH14 could form N–RNA in the presence of N^0^–P_NTD_ but not SH7 (Fig. S2, *A–C*). We also examined three other terminal genome sequences (Le or Tr) of 14-nt RNAs (Le14, LeC14, and TrC14), all of which could form N–RNA complexes. After careful examination of these results, it seemed that Tr14 and LeC14 had more efficient formation of N–RNA than TrC14 and Le14 (Fig. 4*B* and Fig. S2, *D–F*). Therefore, we decided to examine the nucleotide selectivity of the RNA in NCLP assembly.

### Nucleotide selectivity of NCLP assembly

**Figure 5.**
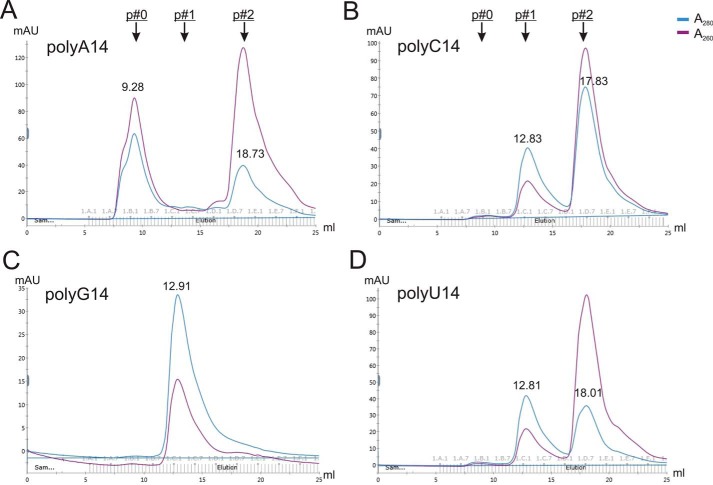
**Nucleotide selectivity of NCLP assembly.** *A–D*, gel filtration SEC profiles of the assembly of N–RNA using 14 nt of the polynucleotide of A, C, G, and U (polyA14, polyC14, polyG14, and polyU14, respectively). Only polyA14 formed peak 0 (N–RNA) but not other types. Peaks 0, 1, and 2 (with *arrows*) are shown at the *top*.

We knew that 14-nt RNA could assemble into NCLPs, but the efficiency varied for different sequences. Therefore, we examined the nucleotide selectivity using 14 nt of the polynucleotide of A, C, G, and U, termed polyA14, polyC14, polyG14, and polyU14, respectively. Surprisingly, only polyA14 formed peak 0 (N–RNA) with purified N^0^–P_NTD_ but not polyC14 or polyU14 (Fig. 5). We noticed that there was no peak 2 for polyG14, and this may be due to the multiple or higher-order G-quadruple (29). These results suggest that A is a preferred nucleotide type to interact with the N protein and assemble into NCLPs.

To avoid self-pairing of RNA, we also tested 14-nt-long mixed AG or CU, AG14 and CU14, respectively. Interestingly, AG14 could stimulate N–RNA formation but not CU14 (Fig. S3, *A* and *B*). To examine whether shorter AG RNA could form NCs, we tested AG7, which also formed N–RNA complexes (Fig. S3*C*). We concluded that purine (A and G) are likely to be the preferred nucleotide types of RNA to stimulate assembly with N^0^–P_NTD_ into N–RNA. Our observation is consistent with other tested NCLPs in related NNS RNA viruses (30, 31).

### Length and sequence dependence of NCLP assembly

To further examine the length and sequence dependence of NCLP assembly, we used nonpreferred nucleotide types. For a nonpreferred nucleotide U, polyU14 did not form NCLPs. Interestingly, when we used a longer RNA with nonpreferred U, polyU28, it gave rise to a nearly complete formation of NCLPs (Fig. S3*D*). We also tested a nonpreferred nucleotide (pyrimidine) pair, C and U, with different lengths. Even though CU14 did not assemble into NCLPs in the presence of N^0^–P_NTD_, a longer CU pair (CU21) could readily form N–RNA (Fig. S3*E*). We conclude that both length and sequence are critical for NCLP assembly but that the longer length of RNA can compensate for formation of NCLPs even though the nucleotide type of RNA is not preferred.

### Minimal length requirements of NCLP assembly

**Figure 6.**
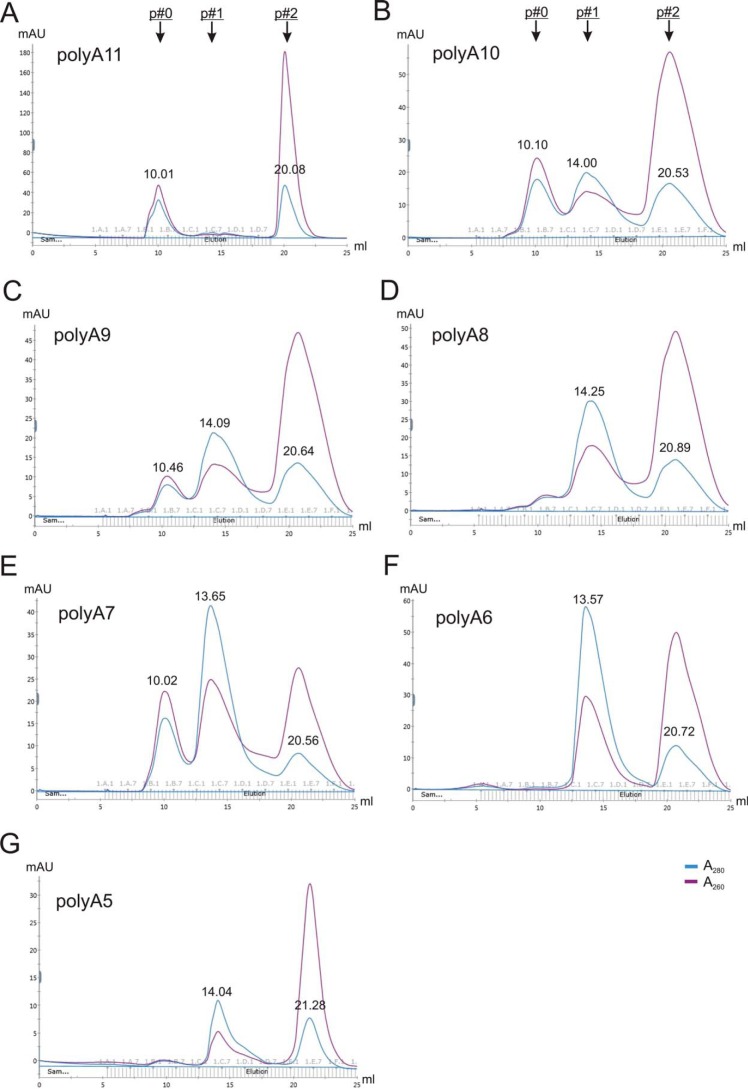
**Minimal length requirements of NCLP assembly.** *A–G*, gel filtration SEC profiles of the assembly of N–RNA using various lengths of the polynucleotide of A: polyA11 (*A*), polyA10 (*B*), polyA9 (*C*), polyA8 (*D*), polyA7 (*E*), polyA6 (*F*), and polyA5 (*G*). Peaks 0, 1, and 2 (with *arrows*) are shown at the top.

Because nucleotide A is a preferred nucleotide type for NCLP assembly, we then used poly(A) to define the minimum length requirements of RNA. We tested the length of poly(A) ranging from 11 nt to 5 nt (Fig. 6). We found that the shortest poly(A) RNA that could stimulate assembly of N–RNA was 7 nt. The results showed that poly6A and poly5A could not assemble into N–RNA (Fig. 6, *F* and *G*). In general, the longer the RNA oligo, the more complete the assembly. For example, polyA11 is better than polyA10 and polyA10 is better than polyA9 to assemble into NCLPs. Interestingly, we found that polyA8 formed a much smaller peak 0 than polyA7 or polyA9. We interpreted this as the potential sticky end of the RNA (1N:7-nt RNA) that interferes with the additional N incorporation into the NCLP assembly. Despite this, the trend of length dependence has also been shown using varying lengths of LeC RNAs. For example, there was a clear trend that LeC10 formed a fraction of N–RNA, and LeC9 formed a less efficient NCLP assembly, but LeC8 failed to form N–RNA (Fig. S4).

Importantly, these results suggest that continuous RNA is not a necessity for NCLP assembly. In contrast to the rule of six in the Paramyxovirus family, the length of RNA is not needed to be a multiple of 7 (7n, n is an integer) to form the N–RNA complex in RSV (32–35). Negative stain EM images of N–polyA14, N–polyA10, N–polyA9, and N–polyA7 showed that they all yielded higher oligomers of N–RNA assembly (Fig. S5). Interestingly, the completeness of the N–RNA assembly of polyA8 is worse than that of polyA9 and polyA7. We interpreted this to be due to the sticky end of one extra nucleotide (A) that hinders efficient NCLP assembly.

### Position specificity of NCLP assembly

**Figure 7.**
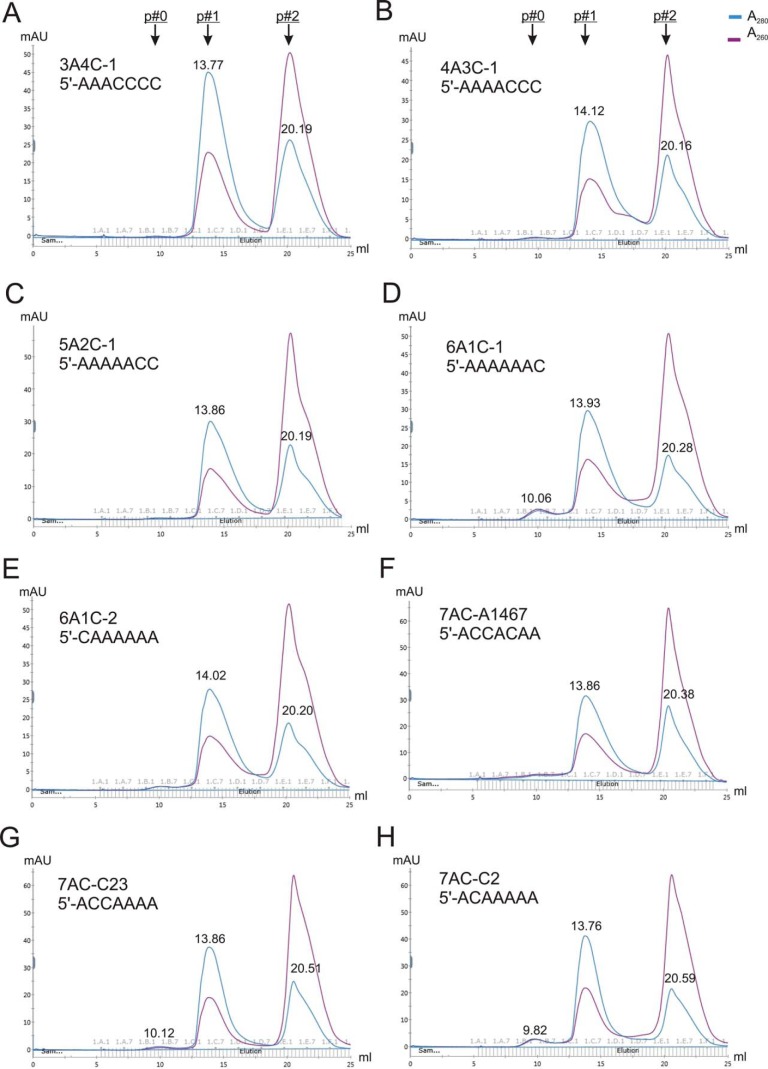
**Sequence specificity of the NCLP assembly.** *A–H*, representative gel filtration SEC profiles of the assembly of N–RNA using scrambled polyA7 with specific positions exchanged to C. We used A as a positive indicator and C as a negative indicator to reveal the importance of a specific position. Additional gel filtration SEC profiles can be found in Fig. S7. Peaks 0, 1, and 2 (with *arrows*) are shown at the *top*.

Now we knew that 7 nt is the minimum length of RNA that could assemble into NCLPs and that N can assemble with any types of RNA when the length is sufficient. We then tested the specificity of the first seven positions of the 7-nt RNA oligos. Because A is a preferred nucleotide and C is not, we used A as a positive indicator and C as a negative indicator to reveal the importance of a specific position. We swapped the A in polyA7 with specific positions converted to C. As expected, the majority of the 7-nt RNAs could not assemble into NCLPs. There are a few exceptions (Fig. 7 and Figs. S6 and S7). For example, the RNAs that contain three, four, or five consecutive As, such as 3A4C-1 (5′-AAACCCC), 4A3C-1 (5′-AAAACCC), and 5A2C-1 (5′-AAAAACC), could not stimulate N^0^–P_NTD_ to assemble into N–RNA (Fig. 7, *A–C*). The SEC profiles of other tested 7-nt RNA oligos showed that most of them failed to assemble into N–RNA (Fig. S6).

We also demonstrated that different positions have different effects on N–RNA assembly. For example, 6A1C-1 (5′-AAAAAAC) could generate a minor peak 0 but 6A1C-2 (5′-CAAAAAA) could not (Fig. 7, *D* and *E*). The results suggest that position 1 is critical because conversion of A to C (at position 1) completely abolished N–RNA assembly, whereas position 7 is less critical because, even with the negative indicator C (at position 7), it could still form peak 0 (N–RNA). We also observed a clear trend when we added a positive indicator (A). For example, 7AC-A1467 (5′-ACCACAA) could not form an N–RNA complex, 7AC-C23 (5′-ACCAAAA) could form a slight peak 0 of N–RNA complex, and 7AC-C2 (5′-ACAAAAA) could generate a minor N–RNA peak 0 (Fig. 7, *F–H*). The results of other 7-nt RNA oligos are shown in Fig. S7, *A–H*. We also tested 8AC-A1 (5′-ACCCCCCC), which did not generate N–RNA assembly (Fig. S7*I*). We also used completely scrambled sequences 7M (5′-MMMMMMM; mixed bases M: A and C) and 7N (5′-NNNNNNN; mixed bases N: A, C, G, and U) (Fig. S7, *J* and *K*). As expected, both 7M and 7N formed a slight peak 0 (N–RNA), indicating that positions and nucleotide types are important for assembly into N–RNA when the length of the RNA is at a minimum. Collectively, these results suggest that, among the first seven positions, positions 1, 4, 5, and 6 have more effects on NCLP assembly than positions 2, 3, and 7.

## Discussion

Much of our knowledge of RSV *cis*-acting signals came from the RSV minireplicon (14, 36). Minireplicons are versions of (−) genomes or (+) antigenomes where marker genes replace viral genes (*e.g.* luciferase). The marker genes are under the control of RSV GS and GE signals. This strategy has been valuable for mapping *cis*-acting signals. However, minireplicons are *in vivo* systems; they do not offer a tractable *in vitro* system for detailed analysis of enzymatic reactions and unstable intermediates. Recently, an *in vitro* RSV RNA polymerization assay using short naked RNA templates for initiation of RNA synthesis was developed (15). In this system, the polymerase acts on short templates in the absence of N protein; however, abortive products are produced at almost every position, along with the template, indicating a nonprocessive interaction (15, 37). Also, the templates do not reach the length threshold (estimated 40–50 nt) required to stimulate the capping and methylation activities of the polymerase (38). Therefore, there is a great need to develop an *in vitro* assay to track NC or NCLP assembly as the cognate template for RNA synthesis by the polymerase.

In contrast to NCs from Paramyxovirus (such as MeV), which follow the rule of six (three bases face in, and three bases face out (19)), for RSV NCs, four of the seven bases face into the surface of the protein, and three of the seven bases face out to the solvent (9). Interestingly, in close examination of the interactions between RNA and the N protein in the structure of RSV NCLPs, it appears that positions 1, 4, 5, and 7 have multiple contacts with the N proteins, whereas positions 2, 3, and 6 have much fewer interactions. These interactions observed in the crystal structure match our position specificity results, except for positions 6 and 7. In the case of 7-nt N–RNA, because the NCLP assembly forms with short RNAs, we speculate that the 5′ end of RNA (position 1) from N (i) may be an essential residue to recruit adjacent N (i-1) to assemble into a higher order of RNA. Positions 3 and 4 are critical to interact with the N (i) protein. However, because the short RNA oligos are not continuous RNAs, the 3′ end of RNA (positions 6 and 7) is not as important. To stack N (i) to its neighboring N (i+1), the 3′ end of short RNA oligo (*i.e.* position 7) may be deployed for primarily stacking effects with RNA (i+1), and this may change how position 6 of the nucleotide is packed with N (i). Of course, these are speculations; further studies of *in vitro* assembled NCLPs are needed to understand the mechanism.

Although most of the *in vitro* assembled NCLPs were similar to the ring-like particles, a small portion appeared as short filaments by negative stain EM (Fig. 4, *D* and *E*, and Fig. S5). When taking a close look at these filaments, most of them are not straight (curved). Judging by the size and overall shape, this observation suggests that the ring-shaped particles likely stack to each other loosely but are not packed into a higher-order filament oligomer. We interpreted this to mean that the RNAs used in this study are short RNA oligos and not continuous to support a higher-order assembly. These observations are consistent with NCLPs from other viruses, such as MeV (39).

As a comparison with previous studies, where the N^0^–P of MeV was reconstituted by fusing N and the P_NTD_ with a cleavable linker (31, 39), in this study, we established a protocol to obtain RNA-free N (N^0^–P_NTD_) of RSV using P_NTD_ as a chaperone without fusing P_NTD_ with N. Therefore, it is easier to separate N and P_NTD_. With purified N^0^–P_NTD_, we successfully assembled NCLPs using short RNA oligos and characterized the NCLPs using gel filtration and negative stain EM.

We defined the length and sequence requirements of RNA for assembly. Using polynucleotides of a specific nucleotide type, we found that A is a preferred nucleotide over C or U. Interestingly, we found that Tr14 (5′-ACGAGAAAAAAAGU) has more efficient N–RNA formation than TrC14 (5′-ACUUUUUUUCUCGU) and that LeC14 (5′-ACGCGAAAAAAUGC) is better than Le14 (5′-GCAUUUUUUCGCGU). We noticed that Tr14 and LeC14 had more preferred A in their sequences than TrC14 and Le14. Importantly, Tr14 and LeC14 are the first sequences that are encapsidated by the N protein during replication by RSV polymerase to synthesize genome (negative-sense) RNA and antigenome (positive-sense), respectively. Therefore, it makes sense that Tr14 and LeC14 can efficiently assemble with N. Nucleotide selectivity could play an important evolutionary role in promoting a desired N–RNA assembly and inhibiting an unwanted N–RNA assembly.

In further examinations, we found that the shortest poly(A) RNA oligo that could stimulate assembly of N–RNA is 7 nt. With specially designed 7-nt RNA, we observed that positions 1, 4, 5, and 6 are essential for N–RNA assembly. In contrast to long filaments of MeV NCLPs, RSV NCLPs generally did not yield long filaments in our experiments. This may be due to the fact that MeV RNAs follow the rule of six, but RSV RNA does not seem to follow such a rule. Alternatively, it may be due to the assembly conditions.

Collectively, our established protocol of an *in vitro* trackable assembly of RSV N–RNA is a powerful tool to dissect the mechanism involved in RNA synthesis. Importantly, we identified an unexpected dependence of N–RNA assembly on the length and sequence of RNA. So far, for many determined structures of NCLP assemblies from NNS RNA viruses, the RNA bases were generically modeled as pyrimidine (uracil or cytosine) to represent the backbone of different nucleotides of random cellular RNA (9, 17–19, 40). Atomic-resolution structures of assembled RNA-specific NCs are needed to fully appreciate the structural basis of the observed length and sequence dependence.

## Experimental procedures

### Molecular cloning of the N^0^–P_NTD_ plasmid

The full-length of the RSV N (residues 1–391) and the P_NTD_ (residues 1–126) were subcloned into a polycistronic plasmid. A His_10_-tobacco etch virus cleavable tag was fused to the N terminus of the N protein, and there was no tag for P_NTD_. The genes encoding for N and P were first amplified separately with an overlap region between their 3′ and 5′ end, respectively. The two DNA fragments were then joined by another round of PCR amplification, which yielded a coexpression construct, His_10_–tobacco etch virus–NP_NTD_.

### Expression and purification of N^0^–P_NTD_

The *E. coli* BL21(DE3) strain was used for protein production. Cell cultures were grown at 37 °C in LB (Miller's Luria Broth) medium until *A*_600_ reached 0.8. The temperature was then lowered to 16 °C, and expression was induced with 0.5 mm isopropyl 1-thio-β-d-galactopyranoside overnight. Cells were lysed by sonication in lysis buffer A (50 mm sodium phosphate (pH 7.4), 500 mm NaCl, 5 mm imidazole, 10% glycerol, and 0.2% NP40), followed by centrifugation. The supernatant was loaded on a cobalt column pre-equilibrated with lysis buffer. The cobalt column was washed with buffer B (50 mm Tris-HCl (pH 7.4), 1 m NaCl, 10% glycerol, and 5 mm imidazole) and buffer C (50 mm Tris-HCl (pH 7.4), 500 mm NaCl, 10% glycerol, and 5 mm imidazole). The protein was eluted from the beads in buffer D (50 mm Tris-HCl (pH 7.4), 500 mm NaCl, and 400 mm imidazole). The protein was further purified by a Q column and then isolated by gel filtration (Superdex 200 column) equilibrated with buffer E (20 mm HEPES (pH 7.4) and 200 mm NaCl).

### In vitro assembly of the N–RNA complex

The purified N^0^–P_NTD_ and RNA oligos were mixed and incubated with a molar ratio of 1:1.5 in buffer E (20 mm HEPES (pH 7.4) and 200 mm NaCl) for 1 h at room temperature. The resulting assembly was analyzed by gel filtration using a Superdex^®^ 200 Increase 10/300 GL (GE Healthcare) pre-equilibrated with buffer E (20 mm HEPES (pH 7.4) and 200 mm NaCl). The N^0^–P_NTD_ (molecular mass = 58 kDa) was usually concentrated to a final concentration of 40–60 mg/ml and stored as the stock. The RNA oligos were purchased from IDT and saved as 0.1 or 1 mm stock. The final concentrations of the protein (N^0^–P_NTD_) and RNA oligos used in the assembly were 13.3 μm and 20 μm, respectively. The sequences of the RNA oligos used in this study are summarized in Table 1. The peak fractions were collected for running the SDS-PAGE gel or making grids.

**Table 1:** **List of RNA oligos** Shown are the name, length, and sequence of the RNA oligos used in this study. The efficiencies of the N–RNA assembly are marked as “−” (negative, no assembly) and “+” (positive, assembly with different degrees).

	Name	Length	Sequence	Efficiency
Proof of principle	Tr45	45	5′-ACGAGAAAAAAAGUGUCAAAAACUAAUAUCUCGUAAUUUAGUUAA	+++++
	Le44	44	5′-AUUUUUUUGGUUUAUGCAAGUUUGUUGUACGCAUUUUUUCGCGU	+++++
	LeC44	44	5′-UGCGCUUUUUUACGCAUGUUGUUUGAACGUAUUUGGUUUUUUUA	+++++
Length preference	Tr21	21	5′-ACGAGAAAAAAAGUGUCAAAA	++
	Tr14	14	5′-ACGAGAAAAAAAGU	+++
	Tr7	7	5′-ACGAGAA	−
	SH21	21	5′-AGUUAAUUAAAAAUAGUCAUA	+++
	SH14	14	5′-AGUUAAUUAAAAAU	++
	SH7	7	5′-AGUUAAU	−
	TrC14	14	ACUUUUU UUCUCGU	+++
	LeC14	14	ACGCGAAAAAAUGC	++
	Le14	14	GCAUUUUUUCGCGU	++
Nucleotide selectivity	PolyA14	14	5′-AAAAAAAAAAAAAA	+++
	PolyC14	14	5′-CCCCCCCCCCCCCC	−
	PolyG14	14	5′-GGGGGGGGGGGGGG	−
	PolyU14	14	5′-UUUUUUUUUUUUUU	−
	GA14	14	5′-GAGAGAGAGAGAGA	+++
	CU14	14	5′-CUCUCUCUCUCUCU	−
Minimal length	PolyA5	5	5′-AAAAA	−
	PolyA6	6	5′-AAAAAA	−
	PolyA7	7	5′-AAAAAAA	++
	PolyA8	8	5′-AAAAAAAA	+
	PolyA9	9	5′-AAAAAAAAA	++
	PolyA10	10	5′-AAAAAAAAAA	++
	PolyA11	11	5′-AAAAAAAAAAA	++
Position specificity	3A4C-1	7	5′-AAACCCC	−
	3A4C-2	7	5′-CAAACCC	−
	3A4C-3	7	5′-CCAAACC	−
	3A4C-4	7	5′-CCCAAAC	−
	3A4C-5	7	5′-CCCCAAA	−
	4A3C-1	7	5′-AAAACCC	−
	4A3C-2	7	5′-CAAAACC	−
	4A3C-3	7	5′-CCAAAAC	−
	4A3C-4	7	5′-CCCAAAA	−
	5A2C-1	7	5′-AAAAACC	−
	5A2C-2	7	5′-CAAAAAC	−
	5A2C-3	7	5′-CCAAAAA	−
	6A1C-1	7	5′-AAAAAAC	+
	6A1C-2	7	5′-CAAAAAA	−
	7AC-A16	7	5′-ACCCCAC	−
	7AC-A17	7	5′-ACCCCCA	−
	7AC-A127	7	5′-AACCCCA	−
	7AC-A137	7	5′-ACACCCA	−
	7AC-A147	7	5′-ACCACCA	−
	7AC-A157	7	5′-ACCCACA	−
	7AC-A167	7	5′-ACCCCAA	−
	7AC-C23	7	5′-ACCAAAA	−
	7AC-C25	7	5′-ACAACAA	−
	7AC-C2	7	5′-ACAAAAA	+
	7AC-A1467	7	5′-ACCACAA	−
	7M	7	5′-MMMMMMM (mixed bases M: A, C)	+
	7N	7	5′-NNNNNNN (mixed bases N: A, C, G, U)	+
	8AC-A1	7	5′-ACCCCCCC	−

### RNA extraction and urea-PAGE gel

The peak fractions of N–RNA were collected after gel filtration SEC. RNA was extracted using phenol–chloroform (Ambion, Thermo Fisher Scientific). Briefly, the N–RNA samples were mixed and vortexed with phenol–chloroform and centrifuged at high speed (15,000 rpm) for 15 min. Then the upper aqueous solution was carefully transferred to 100% isopropanol, inverted, and stored at −80 °C overnight. After high-speed centrifugation at 15,000 rpm for 15 min, the supernatant was discarded. Then the pellets were washed twice by adding 70% EtOH and centrifuged. Then the RNA samples were air-dried and dissolved in nuclease-free water upon use.

### Negative stain EM

Samples were prepared on continuous carbon films supported by 400-mesh copper grids (Ted Pella). 4 μl of the sample was applied to a freshly glow-discharged grid, blotted to a thin film with filter paper, and immediately stained with 1% (w/v) uranyl formate. EM was performed using an FEI Talos L120C electron microscope operating at 120 keV and equipped with an FEI Ceta 4k x 4k charge-coupled device camera. Micrographs were collected at nominal magnifications of ×73,000 (1.97 Å/pixel). The images were acquired at a defocus value of −1.2 ∼ −2.0 μm and electron doses of ∼25 e−/Å2. 2D class averages of negative stain EM micrographs were processed using EMAN2 (41).

## Author contributions

Y. G., D. C., and B. L. conceptualization; Y. G., H. M. A., A. S., M. K., and T. R. data curation; Y. G., D. C., and B. L. formal analysis; Y. G. validation; Y. G., D. C., H. M. A., S. H., C. O., M. K., and T. R. investigation; Y. G., D. C., and B. L. methodology; Y. G. and B. L. writing-original draft; B. L. supervision; B. L. funding acquisition; B. L. visualization; B. L. project administration; B. L. writing-review and editing.

## Supplementary Material

Supporting Information

## Footnotes

2 The abbreviations used are:

NNS: nonsegmented negative-sense
RSV: respiratory syncytial virus
N: nucleoprotein
NC: nucleocapsid
GS: gene start
GE: gene end
Le: leader
Tr: trailer
TrC: trailer complementary
NCLP: nucleocapsid-like particle
P: phosphoprotein
NTD: N-terminal domain
CTD: C-terminal domain
MeV: measles virus
SEC: size exclusion chromatography
nt: nucleotide
AU: absorbance unit.
