# Supplementary material for: In vitro trackable assembly of RNA-specific nucleocapsids of the respiratory syncytial virus
Source: J Biol Chem. 2019 Dec 10;295(3):883–95. doi: 10.1074/jbc.RA119.011602 (PMC6970927; doi:10.1074/jbc.RA119.011602)
Supplement: Supporting Information [file supp_295_3_883__index.html]

In vitro trackable assembly of RNA-specific nucleocapsids of the respiratory syncytial virus — In vitro trackable assembly of the RSV nucleocapsids — In vitro trackable assembly of RNA-specific nucleocapsids of the respiratory syncytial virus — In vitro trackable assembly of RSV nucleocapsids — Supporting Information 

# *In vitro* trackable assembly of RNA-specific nucleocapsids of the respiratory syncytial virus

## Supporting Information

- Supporting Information (to be published online) - Supporting information of the manuscript.
