# Supplementary material for: In vitro trackable assembly of RNA-specific nucleocapsids of the respiratory syncytial virus
Source: J Biol Chem. 2019 Dec 10;295(3):883–95. doi: 10.1074/jbc.RA119.011602 (PMC6970927; doi:10.1074/jbc.RA119.011602)
Supplement: Supporting Information [file supp_RA119.011602_156484_1_supp_435409_q1lbpv.pdf]

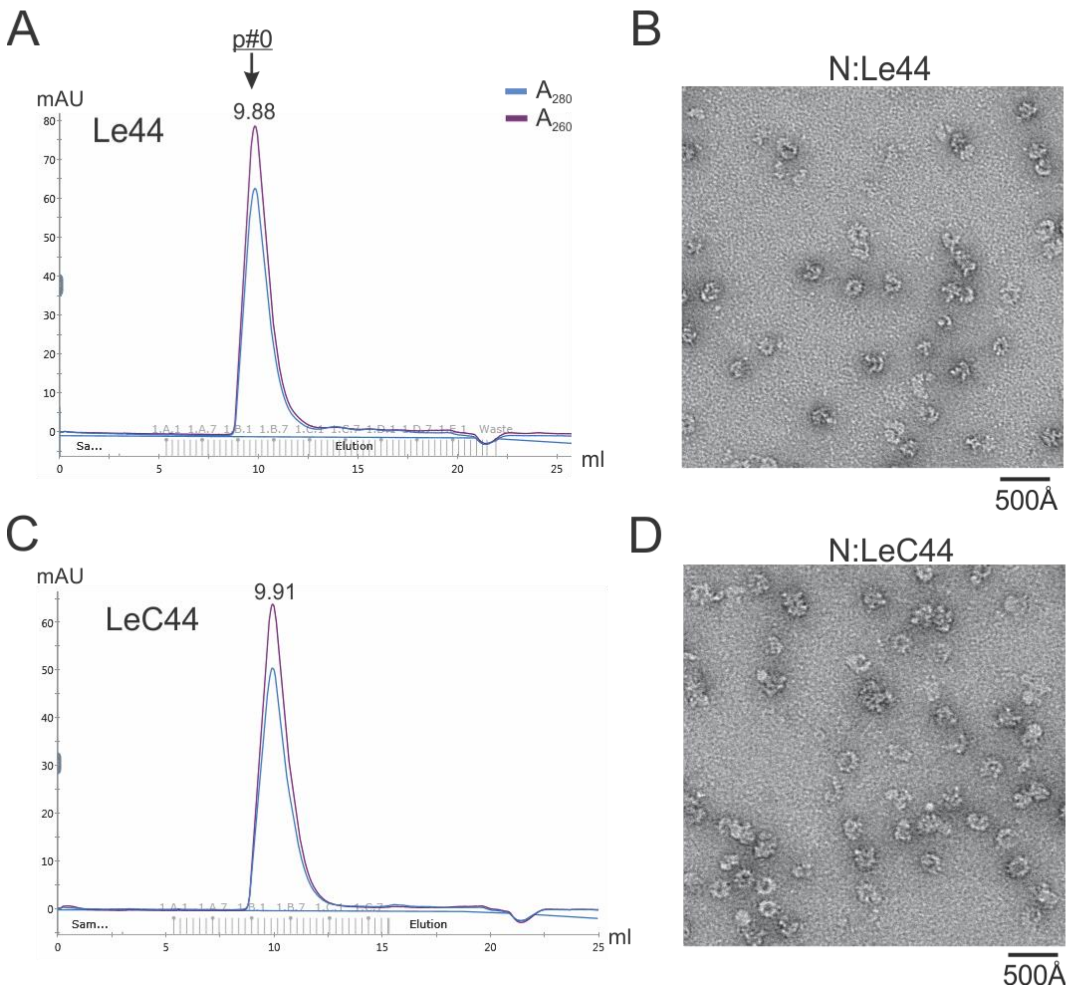

Figure S1: *In vitro* assembly of N:RNA using RSV-specific RNA oligos. (A,C) The gel filtration SEC profile of the assembly of N:RNA, N:Le44 (A), and N:LeC44 (C). Peaks #0, #1, and #2 with arrows indicate the N:RNA, N, and RNA, respectively. **(B,D)** Representative negative stain EM images of assembled N:RNA complex, N:Le44 (B), and N:LeC44 (D). The ring-like shape particles with spikes are the NCLPs (N:RNA).

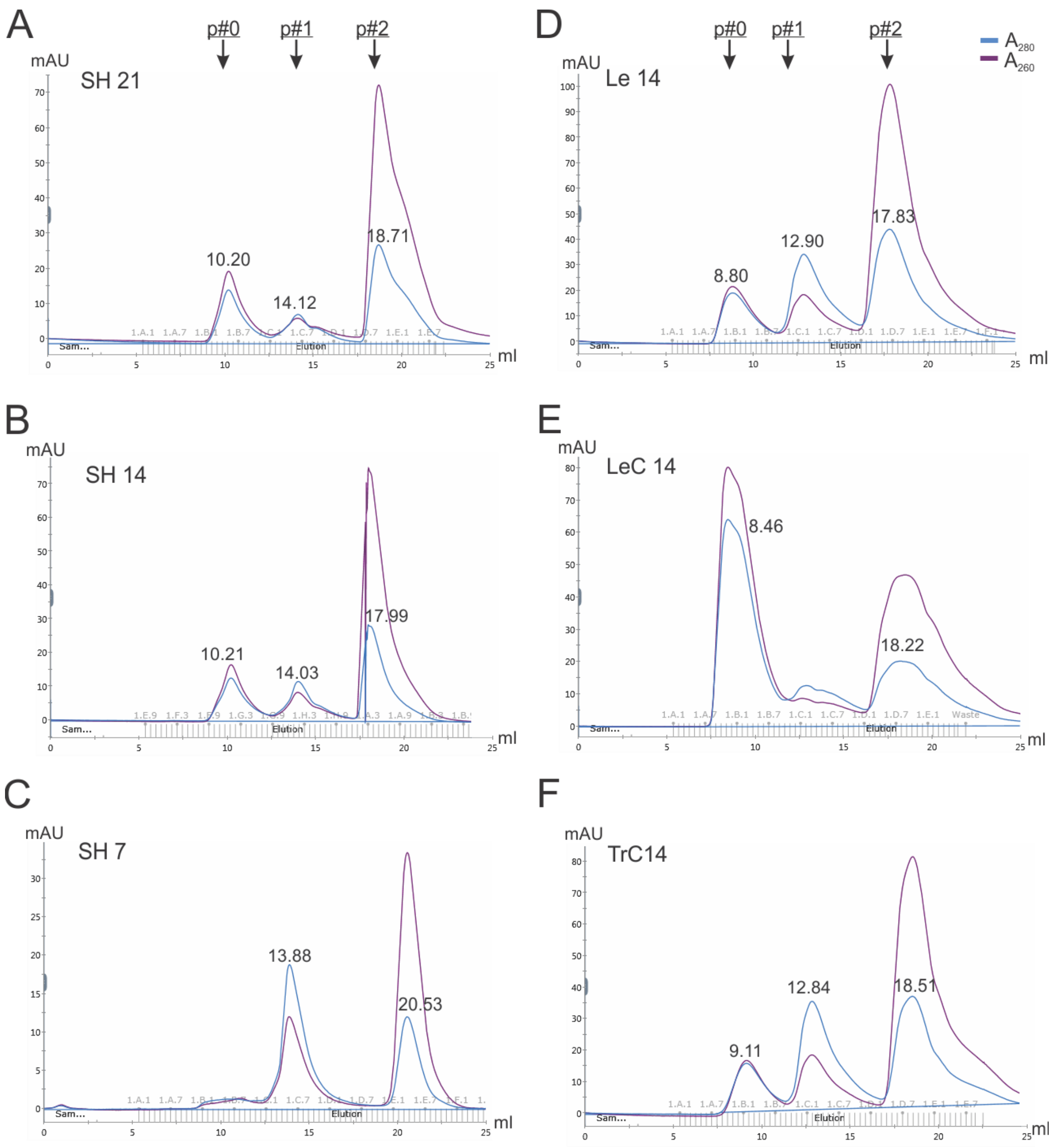

Figure S2: Length dependence of the N:RNA assembly. (A-C) The gel filtration SEC profiles of the assembly of N:RNA using 21-nt, 14-nt, and 7-nt of SH sequence (SH21, SH14, and SH7), respectively. (**D-E**) The gel filtration SEC profiles of the assembly of N:RNA using 14-nt leader or trailer sequences, Le14, LeC14, and TrC14, respectively.

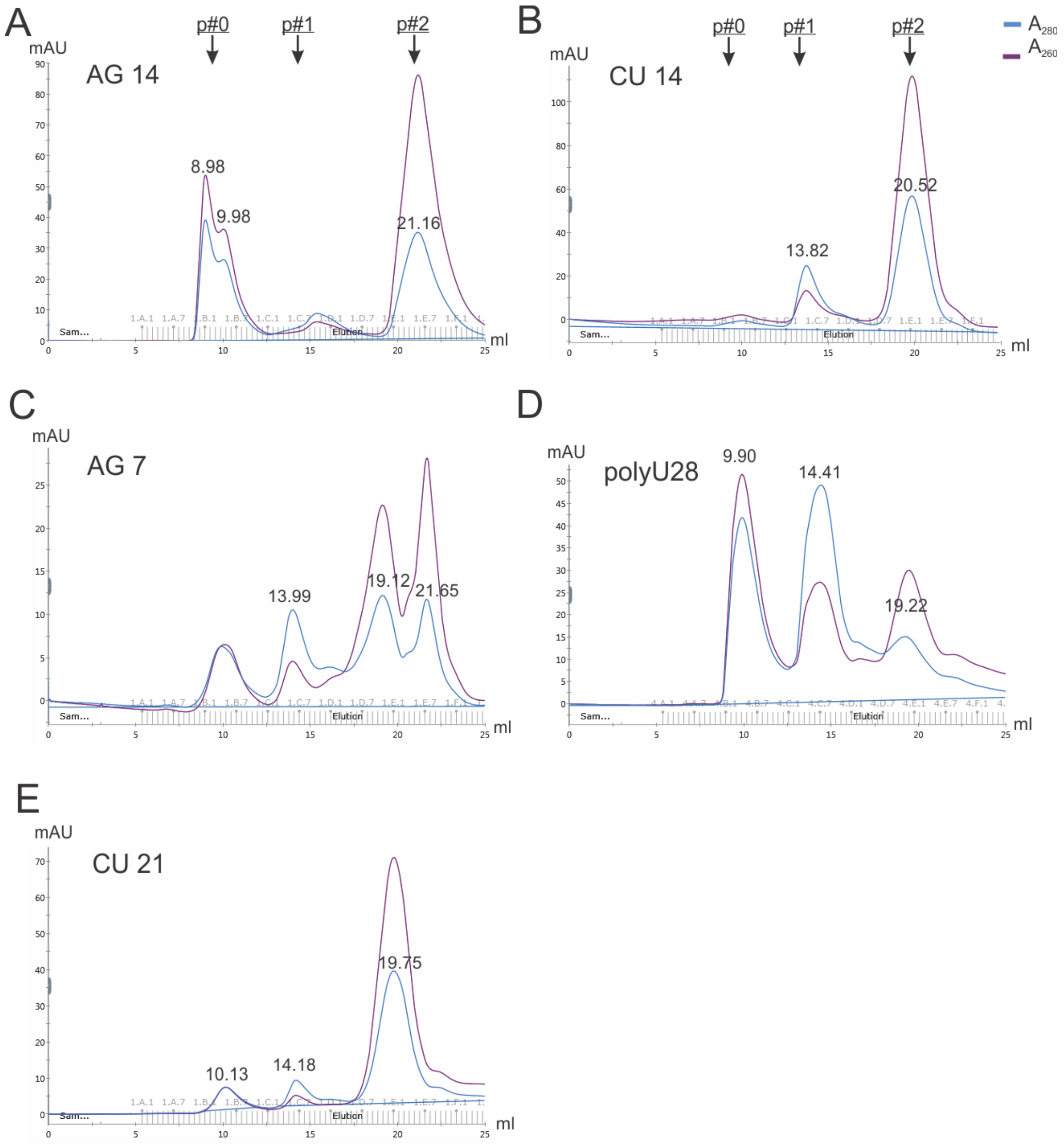

Figure S3: Nucleotide selectivity of the NCLP assembly. (A-E) The gel filtration SEC profiles of the assembly of N:RNA using AG 14, CU 14, AG 7, polyU28, and CU 21, respectively.

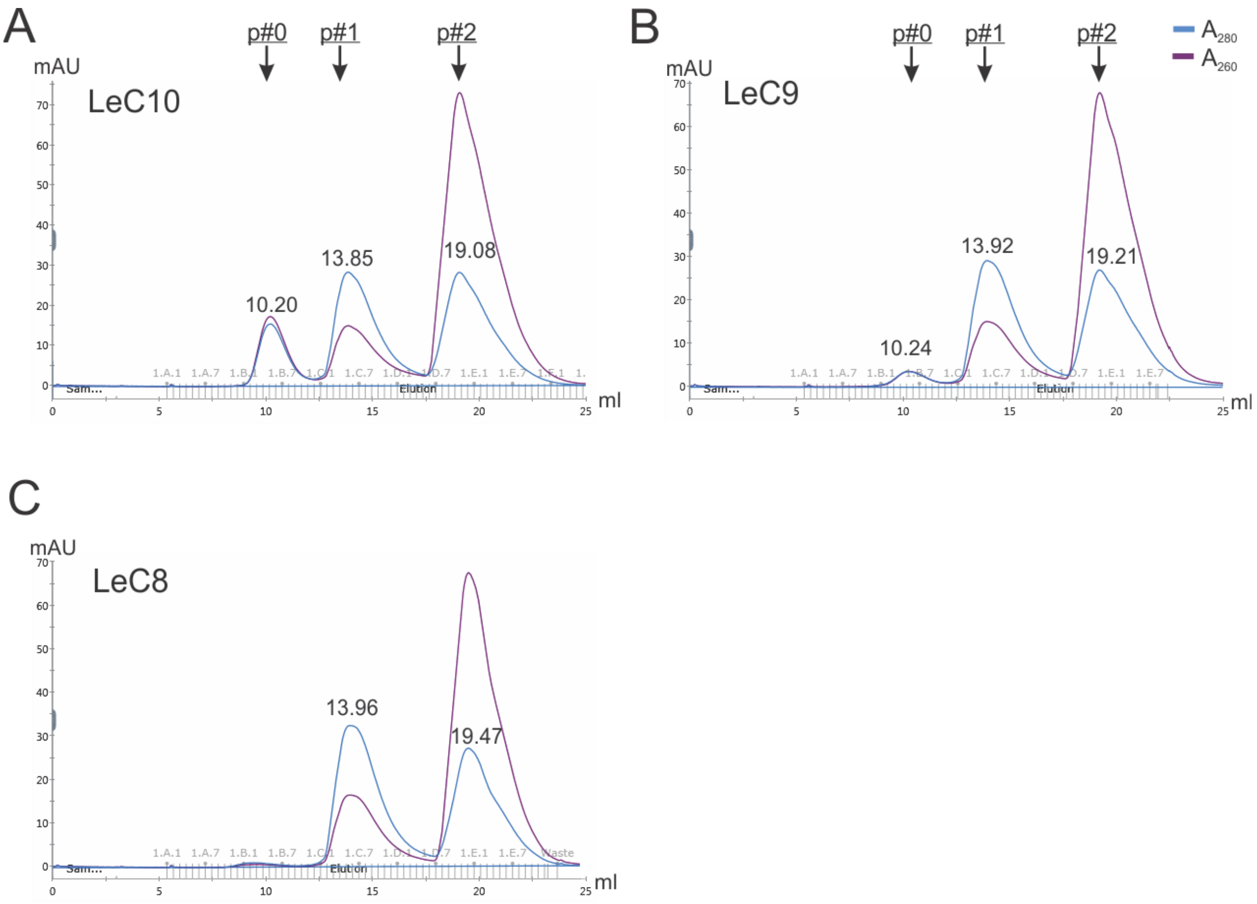

Figure S4: Minimal length requirements of the NCLP assembly. (A-C) The gel filtration SEC profiles of the assembly of N:RNA using LeC10, LeC9, and LeC8, respectively.

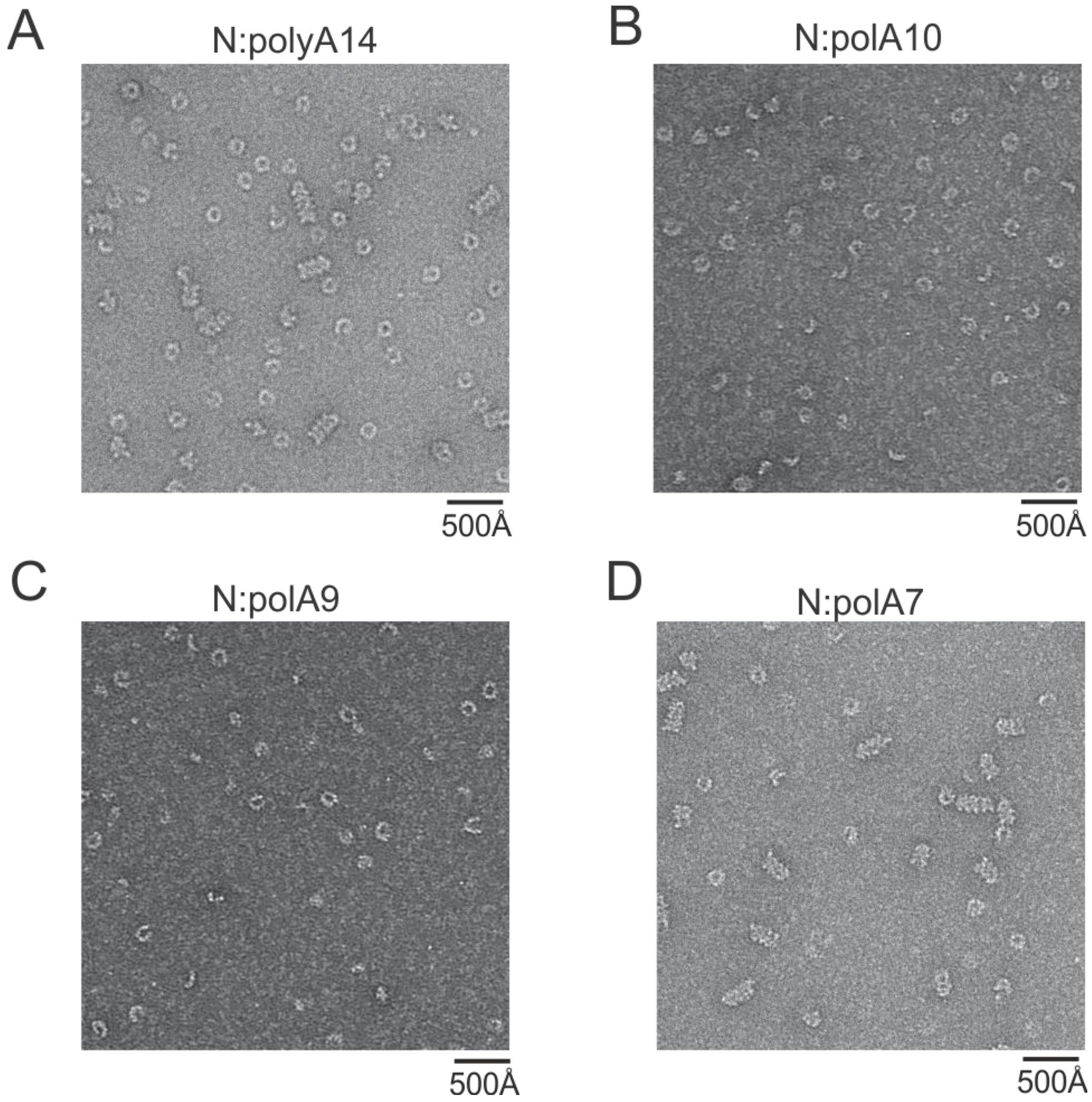

Figure S5: Representative negative stain EM images of assembled N:RNA complex. (A-D) N:polyA14 (**A**) N:polyA10 (**B**), N:polyA9 (**C**), and N:polyA7 (**D**), respectively.

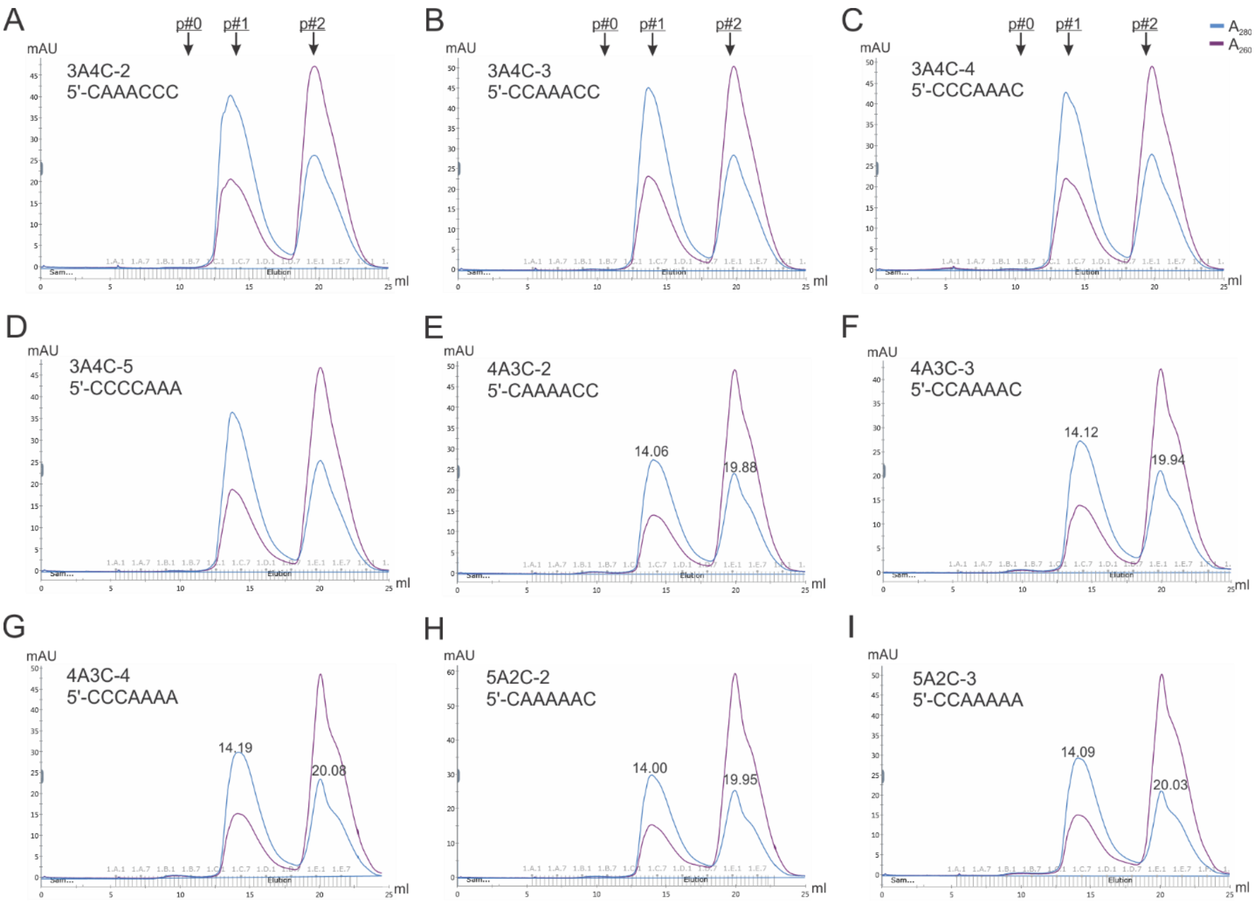

Figure S6: Sequence specificity of the NCLP assembly. (A-I) Representative gel filtration SEC profiles of the assembly of N:RNA using scrambled polyA7 with specific positions exchanged to C.

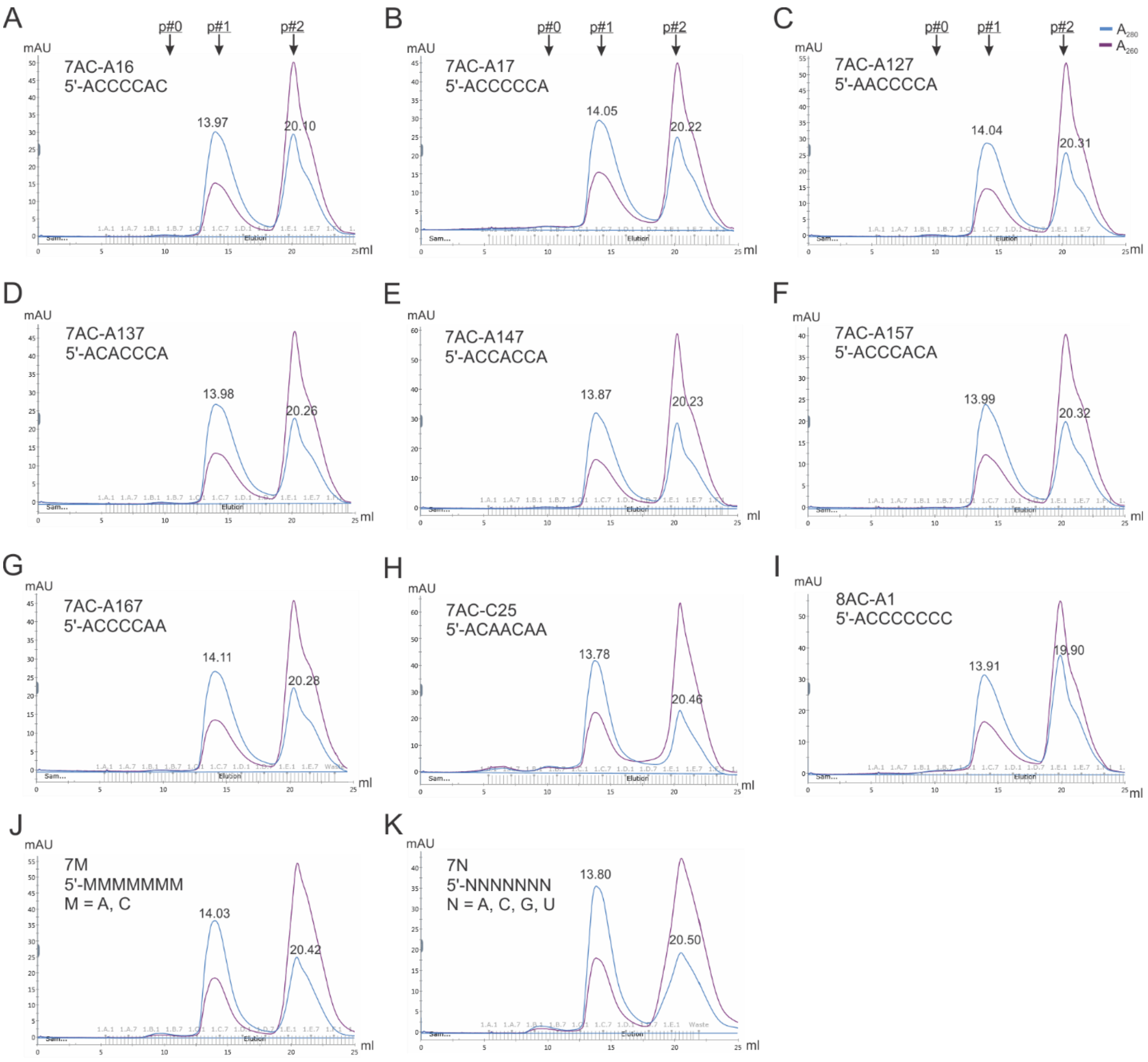

Figure S7: Sequence specificity of the NCLP assembly. (A-K) Representative gel filtration SEC profiles of the assembly of N:RNA using scrambled polyA7 with specific positions exchanged to C.
